# Supplementary material for: Comparison of anti-HCV combined with HCVcAg (Elecsys HCV Duo immunoassay) and anti-HCV rapid test followed by HCV RNA analysis using qRT-PCR to identify active infection for treatment
Source: PLoS One. 2024 Nov 18;19(11):e0313771. doi: 10.1371/journal.pone.0313771 (PMC11573151; doi:10.1371/journal.pone.0313771)
Supplement: Attachment — Submitted filename: Reviewer Comments to Authors PONE-D-24-35203 (Ahmed Ismail).pdf [file pone.0313771.s001.pdf]

Comparison of anti-HCV combined with HCV cAg (Elecsys® HCV Duo immunoassay) and anti-HCV rapid test followed by HCV RNA analysis using qRT-PCR to identify active infection for treatment

Comments to Authors

Reviewer Comments to Authors: Manuscript No.: PONE-D-24-35203

Comparison of Elecsys® HCV Duo assay versus Anti-HCV RDT followed by qRT-PCR to Identify active infection

I went through the manuscript, and after thoroughly reviewing and evaluating the scientific attributes and level of importance of the manuscript, I am hereby sending the following comments to the Authors:

The authors' aim was to compare the Elecsys® HCV Duo immunoassay and the traditional two-step process, which involves initial anti-HCV antibody testing followed by quantitative real-time polymerase chain reaction (qRT-PCR) for detecting active hepatitis C infections. Additionally, the study aimed to evaluate the relationship between HCVcAg and qRT-PCR assay in different genotypes.

Comments to Authors:

1. While the study holds significant value, especially within the context of the global effort to eliminate HCV, the authors should consider the following points:

A. <u>Introduction:</u>

i. In line 69: include a reference on the adoption of HCV core antigen testing by WHO recommendation. This will provide credibility regarding the global significance of core antigen testing.

ii. Provide a brief mention of the national guidelines for HCV testing in Thailand and whether HCV core antigen testing has been adopted in these guidelines. This will help ground the study in the context of local diagnostic practices and highlight the relevance of your findings for Thailand's national guidelines.

iii. Elaborate on the advantages of using the assay of anti-HCV combined with HCV cAg in real-world settings, particularly its implications for reducing costs and turnaround time in HCV screening and diagnostics.

B. <u>Materials and Methods:</u>

i. Flow and Clarity: Improving the flow of the methodology would significantly enhance its clarity and make it easier for readers to follow. A more logical structure would ensure that each step of the study is clearly outlined, facilitating comprehension. For example, as noted below, you could improve the transition between the previous and current study methods for better readability.

ii. Serology assay in the Laboratory assessment Section: Reorganize this section by starting with information on how the samples were categorized into active infection and resolved infection groups, referencing the use of Bioline™ HCV RDT from the previous study (Figure 1). Then,

follow this by explaining how the current study employed the Elecsys® Duo assay (anti-HCV & HCV cAg) to further assess these groups. This approach will make it easier for readers to understand how the two testing methods are related and integrated into the study.

iii. **Sample Processing**: More detail should be mentioned on how samples were processed, in particular in regard to the handling of frozen samples and any potential effects on antigen or RNA stability.

iv. **Line 169 - Reference Standard**: In line 169, it is mentioned that qRT-PCR was used as the reference standard to evaluate the Duo-assay's performance in terms of sensitivity and specificity. While this is correct for the HCV core antigen (HCV cAg) component of the assay, it is not correct for the anti-HCV component. For anti-HCV testing, a more specific assay is required for confirmation, such as the Line Immunoassay (LIA). Typically, tests like the INNO-LIA HCV Score are used to confirm the presence of HCV antibodies with greater specificity. Therefore, it is important to clarify what reference standard was used to evaluate the performance of the Duo anti-HCV component. If a Line Immunoassay or a similar confirmatory test was utilized, this should be explicitly mentioned. Doing so will enhance the accuracy of the methodology and ensure that the reported sensitivity and specificity values for the anti-HCV assay are appropriately validated against the correct reference standard.

C. Results:

i. The result part needs improvement in terms of clarity and interpretation. By Embedding brief interpretations within the results to highlight the clinical or practical significance of the findings, without waiting for the discussion section.

ii. Line 191 and 194: In lines 191 and 194, you refer to "the Duo and anti-HCV modules." For clarity and consistency, I suggest changing this to "the Duo/anti-HCV modules" throughout the manuscript wherever the Duo and anti-HCV are mentioned. This will provide a clearer and more concise reference to the different components of the assay, making it easier for readers to follow.

iii. Line 197: In line 197, you mention "To validate the sensitivity of the Duo and Elecsys® anti-HCV assays." If the study only used the Elecsys® Duo assay (which includes both anti-HCV and HCV cAg components), and not a separate Elecsys® anti-HCV assay, please revise this sentence for accuracy. Instead, it should read: "To validate the sensitivity of the Elecsys® Duo (anti-HCV) assay." This will ensure that readers are not confused about whether multiple assays were used, and it accurately reflects the methodology. If both the Duo and separate anti-HCV assays were used, make sure this distinction is clearly outlined.

D. Discussion:

i. While the Elecsys® Duo assay demonstrates 100% sensitivity for detecting anti-HCV antibodies, its 70% sensitivity for detecting HCV core antigen (cAg) indicates that 30% of active infections might be missed if relying solely on cAg detection. This limitation should be explicitly acknowledged in the manuscript, particularly in the context of resource-limited settings where access to HCV RNA testing may be restricted or unavailable. In these settings, the inability to detect all active infections could impact clinical decision-making, delaying treatment initiation for some patients. Discussing the importance of complementary testing (e.g., follow-up with RNA testing) where possible, or alternative strategies to manage false negatives, will make the discussion more balanced and practical.

ii. Detailed discussions on clinical applicability will enhance the quality of the manuscript.

E. <u>Informed consent statement:</u>

i. The ethical considerations concerning anonymized data usage should be emphasized more.

F. <u>Funding:</u>

i. To further reduce any potential conflict of interest (COI) concerns, it is recommended that you include the following statement in the Funding section: "The funders had no role in study design, data collection and analysis, decision to publish, or preparation of the manuscript." This statement will reassure readers and reviewers that the research was conducted independently and that the funding sources did not influence any aspect of the study's design, execution, or reporting. This addition will enhance the transparency of the manuscript and ensure that any concerns regarding objectivity are adequately addressed.

G. <u>Figures and Tables:</u>

i. Provide a legend for each figure and table to guide the reader in interpreting the data, especially for less familiar readers
ii. The tables should be revised to be clearer and more informative.

H. <u>Minor Comments:</u>

i. Review the manuscript for minor grammatical issues. Shorter, clearer sentences would improve readability.
ii. Consistency in terminology (e.g., using "Elecsys® Duo assay" versus "Duo-assay") should be maintained throughout the text.
